# Gender Participation and Performance in Boccia International-Level Events

**DOI:** 10.3390/jfmk10010087

**Published:** 2025-03-06

**Authors:** Cátia C. Ferreira, José M. Gamonales, Jesús Muñoz-Jiménez, Mário C. Espada

**Affiliations:** 1 Training Optimization and Sports Performance Research Group (GOERD), Faculty of Sport Science, University of Extremadura, 10005 Cáceres, Spain; catia.ferreira@ese.ips.pt (C.C.F.); martingamonales@unex.es (J.M.G.); suliwan@unex.es (J.M.-J.); 2 Instituto Politécnico de Setúbal, Escola Superior de Educação, 2914-504 Setúbal, Portugal; 3 Faculty of Education and Psychology, University of Extremadura, 06006 Badajoz, Spain; 4 Centre for the Study of Human Performance (CIPER), Faculdade de Motricidade Humana, Universidade de Lisboa, Cruz Quebrada-Dafundo, 1499-002 Lisboa, Portugal; 5 Comprehensive Health Research Centre (CHRC), Universidade de Évora, 7004-516 Évora, Portugal; 6 SPRINT Sport Physical Activity and Health Research & Innovation Center, 2001-904 Santarém, Portugal; 7 Life Quality Research Centre (CIEQV-Setúbal), Escola Superior de Educação, 2914-504 Setúbal, Portugal

**Keywords:** adapted sports, human behavior, equity, inclusion, male, female

## Abstract

**Background/Objectives:** Boccia is an attractive and growing adapted sport. For approximately 30 years, this parasport was played together by male and female athletes, a fact that recently changed, to our best knowledge, without scientific support. Hence, this study aimed to analyse the relationship between gender participation and performance in Boccia international-level events. **Methods:** For data collection, four specific international-level Boccia events between 2012 and 2018 were selected as partials were available in the official competition websites (2708 partials, which represent a total of 32,496 ball throws). **Results:** We found that partials won by male athletes systematically increased between 2012 and 2018 but tended to stabilize between 2017 and 2018, contrary to females, with a growing trend from 2016 onwards. No differences were observed, considering the players’ gender and the type of partials (adjusted, balanced, and unbalanced) in the Boccia classes BC1, BC2, and BC3. In BC4 differences were found, but with little variance or low association level (Cramer’s Phi coefficient of 0.114). **Conclusions:** The results emphasize that based on performance, both men and woman can play Boccia together. Although, if the focus of separating genders in Boccia is toward growing and effective female participation and equal success and reward opportunities, this study highlights as a good perspective aiming regular practice of physical activity, exercise, and sport in people with disabilities, promoting their quality of life.

## 1. Introduction

On 1 January 2016, the 17 Sustainable Development Goals (SDGs) of the 2030 Agenda for sustainable development were officially implemented, previously adopted by world leaders in September 2015 at a historic United Nations (UN) Summit. The fifth, “gender equality”, is a global challenge society faces. Sustainability is a complex concept and sport, a society pillar related to inclusion, equality, participation in social life, tolerance, and acceptance of differences. The UN 2030 agenda, aiming at transforming our world, highlights gender equality, empowerment, and self-determination of all women and girls as strategic themes in modern society.

Regular physical activity (PA) is acknowledged as an important part of a healthy and balanced life, essential for maintaining good health, preventing disease [1,2], and contributing to global well-being. The benefits of PA are numerous and play a significant role in physical and mental health throughout the life span [3,4]. Numerous aspects of health are now well-known and generally accepted [5,6,7]. Notably, PA participation is associated with decreased morbidity and mortality in adulthood [8]. Moreover, participation in sports is closely related to other SDGs, at a time that is necessary to guarantee full access to the right to health for all through an effective approach to PA and sports, with particular attention to special populations that are more exposed to risk factors for health and well-being.

Adapted sports emerged in the 1940s but have been introduced as rehabilitation for ex-war combatants. Nowadays, adapted sports are practiced by all the population, with and without disability, from pre-school to the aging. Games are as old as human civilization itself. For centuries, they were used to entertain both children and adults [9]. In scientific literature, there is a diversity of studies related to PA and sports for people with disabilities [10,11,12]. Boccia is a sport that is particularly well-suited for individuals with severe physical disabilities and is inclusive of all disability groups. Currently, it is one of the fastest-growing adapted sports in the world.

At first, Boccia was designed specifically for individuals with Cerebral Palsy (CP), but it was later expanded to include athletes with a variety of physical disabilities [13], with the aim of the game to improve the quality of life (QoL) of its participants and facilitate their integration into society [14]. Through its emphasis on strategy, teamwork, and social interaction, Boccia fosters emotional regulation, empathy, and interpersonal skills, contributing to the development of emotional intelligence [15]. It is an indoor sport that is inclusive of all genders, ages, and abilities, as noted by Coutinho and Acosta [16], and it is played on a court with two sides (of one or two players each). Each side has six balls (red or blue), and players must throw these balls, using a method determined by their classification, out into the playing zone and as close as possible to the jack (the white ball) on a playing hard surface court measuring 12.5 × 6 m.

Boccia is safe to perform and even easier to improve in [17], characterized as a parasport of strategy and precision, played by athletes in wheelchairs [18]. Boccia is played by people with disabilities, but practiced by the whole population, from children in schools to the elderly, with social and rehabilitation objectives. The classification system of individual play, defined as functional classification (FC), comprises four categories: BC1—athletes with CP who have restricted trunk movement and poor sitting balance but can throw the ball, usually overhand; BC2—athletes with CP who have greater sitting balance than BC1 athletes and are usually able to pick the ball up from the floor and throw either overhand or underhand; BC3—athletes with CP who are unable to hold and release a ball and therefore may use a ramp and an assistant; and BC4—athletes who have a severe physical disability with a diagnosis other than CP, such as progressive muscular dystrophy [19]. In summary, BC1, BC2, and BC4 players move in a wheelchair and do not receive assistance during the throw or resort to a ramp, contrary to BC3.

There is a great lack of knowledge about the different sports modalities available for people with disabilities, especially those that are specific (e.g., Boccia, slalom, and goalball) [20]. Very little research has reported scientific evidence related to Boccia athletes, one of the recent conclusions was that there is a great space and need for further research, particularly regarding “Coaching Science”, to improve training conditions and optimize sports performance [21], with previous studies focusing on the technical, biomechanical, and learning aspects of Boccia throwing [22,23,24,25]. This parasport was practiced for many years as a leisure activity, only introduced at the Stoke Mandeville and New York 1984 Paralympic Games (PG) as a competitive sport, played separately by men and women in this unique edition, and mixed from Seoul 1988 to Tokyo 2020 (which took place in 2021 due to the COVID-19 pandemic).

More recently, during the Rio de Janeiro 2022 World Boccia Championships (WC) and the Paris 2024 PG, Boccia was again separated by men and women, approximately 30 years after being introduced in the PG, a decision that significantly changed issues such as the qualification model for participation in Boccia sporting events, the number of participants per gender in competition, and the possibility of recognition for performance (podiums, medals, media coverage, etc.). In our view, it could also influence the game in pairs and teams, all of these, to our best knowledge, without scientific support.

Additionally, the regular practice of PA, exercise, and sport in people with disabilities is related to regulatory measures determined by organizations, which can influence motivation to practice adapted sports, and in this sense, on opportunities to promote (QoL) in people with disabilities. Previous research demonstrated that only 40% of women take part in the minimum recommended amount of PA [26,27], and in general, people with disabilities are in poorer health than the general population [28,29,30]. People with different disabilities such as CP often face significant barriers to participating in PA [31,32,33], and in particular, women with physical disabilities often face unique and multiple challenges, including social, psychological, and physical barriers [34]. These facts are a challenge in modern society, and particularly regarding adapted sports such as Boccia. Hence, this study aimed to analyse the relationship between gender participation and performance in Boccia international-level events. We hypothesized that male participation is higher compared to female in individual Boccia classes at international-level events, but no gender differences exist regarding performance.

## 2. Materials and Methods

### 2.1. Design

The design of this study presents a descriptive and associative strategy [35], to identify whether there are differences between two or more study variables. For this purpose, the official results of the 2012 and 2016 Boccia PG editions, European Championships (EC), and WC (2017 and 2018, respectively), were retrieved from the official competition websites and worldboccia.com. For analysis, the results in game partials (an individual segment of a game based on ball throws) from the start of international-level events (preliminary phase) to finals were considered and registered. Games decided by tie-break were excluded from the analysis.

The four specific international-level Boccia events were selected because results from partials were available, contrary, for example, to Tokyo 2020, in which only game results were available (the sum of the partials, but not their description). The same was found regarding the 2013 EC and 2014 WC. Each game of the individual classes BC1, BC2, BC3, and BC4 consisted of four partials. The result in each of the halves (partial) depended on the performance in the six throws that each player made. At the end of each partial, the balls of the same color closest to the white were counted. The sum of all the partials dictated the winner. In short, the result of each game depended on the 24 throws made by each player, or globally, 48 ball throws.

### 2.2. Variables

The definitions of each study variable and its categories were proposed in previous research involving a group of experts in sports for people with disabilities [36,37,38]. These variables used for the analysis of performance indicators were based on previous research methodology [39]. All were categorized numerically to facilitate their registration and subsequent statistical analysis.

Since there is no previous research on this particular theme of the game in Boccia, we codified the variables related to the results in the partials and game results [40,41]. Table 1 shows the variables’ type of partial nomenclature and respective point intervals.

Considering the purpose of this study, the variables considered for analysis are as follows: (i) Functional classification (FC): BC1, BC2; BC3, BC4; (ii) Competitive event: PG 2012, PG 2016, EC 2017, and WC 2018; (iii) Gender: male and female; and (iv) Type of partial: adjusted, balanced, unbalanced.

### 2.3. Procedures

A process of training and familiarization with data collection was carried out, to guarantee data reliability. The training and reliability procedure with an experienced observer PhD in Sports Sciences had four stages [37]: (1) Preparatory stage, (2) Selection stage of the coders, (3) Training stage, and (4) Reliability stage. Inter-observer reliability was analysed to guarantee the quality and validity of the data collection for subsequent statistical analysis and to ensure that relevant conclusions could be drawn. This process was previously used in scientific literature in different sports contexts, such as grassroots football [42].

Reliability assessments included calculations of the intraclass correlation coefficient (ICC) [43]. The ICC values of <0.5 were considered indicative of poor reliability, values of 0.5–0.75 were indicative of moderate reliability, values of 0.75–0.90 were indicative of good reliability, and values of >0.90 suggested excellent reliability [44]. Overall, 2708 Boccia partials were analysed, which represents a total of 32,496 ball throws by Boccia players, since, as previously indicated, every player throws the ball six times in each partial. The number of partials considering the different Boccia FC is presented in Table 2.

### 2.4. Statistical Analysis

Data is presented as mean (M) and standard deviation (SD). The Chi-square (χ^2^) was used [44], to assess the level of association between the variables using Cramer’s Phi coefficient (φc) [45]. The level of association was interpreted according to Crewson’s proposal: Small (<0.100), Low (0.100–0.299), Moderate (0.300–0.499), and High (>0.500).

For the interpretation of the degree of association of the variables studied, the Adjusted Standardized Residuals (ASR) was used [46]. The software used for the analysis was the Statistical Package for the Social Sciences (v29.0.2.0, IBM Corp., Armonk, NY, USA). The significance level was set at *p* < 0.05.

## 3. Results

**Figure 1:**
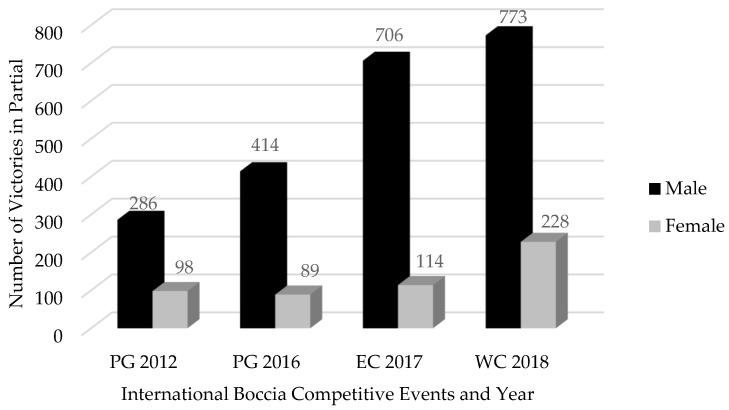
Partials won by gender in four international-level Boccia events between 2012 and 2018.

Figure 1 depicts the distribution of partials won by different gender players in the four international-level Boccia events considered for analysis.

High levels of reliability (ICC > 0.84 and <0.92) were observed. A total of 2179 partials were won by male Boccia players and 529 by female cohorts. Table 3 presents the descriptive statistics of the partials won considering each Boccia FC and gender, based in the analysed international-level events.

We can observe that the number of partials won by individual male players was similar between 2012 and 2018 in Boccia international-level events, with a minimum of 509 and a maximum of 577 (in BC1 and BC2, respectively). On the other hand, the number of partials won by female players was visibly lower compared to males, with a minimum of 91 (in BC1, same as in the male cohorts), and a maximum of 188 (in BC3).

Considering all the played partials and the different FC, we should highlight that the highest percentage of partials won by female players was 25.27% and the lower value in males 74.73% in the same FC, in both cases values associated with BC3, players who perform with ramp and sports assistant. The influence of gender in the Boccia type of partials is shown in Table 4.

No differences were observed in BC1, BC2, and BC3, contrary to the found regarding BC4, despite the observed little variance, Cramer’s Phi coefficient of 0.114, a low association strength, according to Crewson [45]. Table 5 describes the analysis of ASR, considering the BC4 individual Boccia FC.

In BC4, we found a high probability of the Boccia partials won by female players, to be considered adjusted (1–2 ball difference), and in contrast, a higher probability of balanced partials in male Boccia players (3–4 ball difference). This is to say that a tendency is observed for the Boccia partials won by females to be closely disputed.

## 4. Discussion

This study aimed to analyse the relationship between gender participation and performance in Boccia international-level events. To our best knowledge, this is the first scientific approach to this topic, at a time when the regulatory framework in Boccia is changing with an impact on gender equality in the parasport. As main findings, we found: (1) Partials won by male athletes systematically increased in international-level events between 2012 and 2018 but tended to stabilize between 2017 and 2018, contrary to female, with a growing trend from 2016 onwards, doubled between 2017 and 2018; (2) Considering all the FC in Boccia, in the four international-level studied events and considering the game partials (2708), we found male victories to be always above 74% in the four FC (totalizing 2179), and in female, a total of 529 partials won; (3) No differences were observed considering the players’ gender and the type of partials in BC1, BC2, and BC3, contrary to the findings regarding BC4 (low association level); and (4) In BC4, there was a high probability of the Boccia partials won by female players to be considered adjusted (1–2 ball difference), and in contrast, a higher probability of balanced partials in male Boccia players (3–4 ball difference).

Physical/sports activities for people with disabilities contribute to their functional independence, improve their physical condition, performance and physical capacity, favor the prevention and correction of deformities and postural defects, reduce stress, and improve self-confidence, emotional states, relationships with others, enjoyment and interest, among other things [47,48]. According to Armstrong and Welsman [49], gender is the difference between females and males biologically, since a person is born and gender differences are one of the factors that affect the ability to move. These authors stressed that boys and girls have slightly different aerobic fitness before puberty and the peak maximal oxygen uptake (VO_2max_) will increase according to certain genders, along with morphological changes driven by age and maturity with time and changes specific to individuals. Considering as an example the hammer throw in elite-level athletes, Pavlović [50] stated that there was a significant difference between male and female finalists in the 2011 Daegu WC, suggesting that differences are associated with the different training processes, as well as morphological profile, motor structure, and anatomy.

Addressing this topic, and focusing on adapted sports, Cavedon et al. [51] indicated that the performance of the field test on wheelchair players (in particular, wheelchair basketball) was affected due to gender differences, proposing that training take into account gender to improve skills. Likewise, previous research highlighted differences in the performance of men and women in goalball [52]. Therefore, in these team-adapted sports associated with specific physical and psychological demands, it was recommended that the training process be carried out based on gender and that the competitive moments consider the separation between male and female athletes. More recently, when analysing the association between serve efficacy and match outcome in sitting volleyball players, it was found that performance in the side-out phase is crucial for competitive success in both male and female volleyball [53].

Nevertheless, in our study, conducted in individual Boccia FCs, no differences were observed considering the players’ gender and the type of partials in BC1, BC2, and BC3, which represent to say that in four international-level Boccia events between 2012 and 2018 and considering 2055 partials and 24,660 ball throws, the type of partial was not influenced by the gender of the Boccia player, even though we all know that the number of male participants in these competitive events tended to be higher compared to the number of female participants. All these FCs (BC1, BC2, and BC3), are associated with athletes with CP, and several studies related to this physical disability show that physical-sports activity can produce benefits for these people. In particular, Sanz and Reina [54] considered that it can improve balance, coordination, speed of movement and reaction time, range of motion, and mechanical efficiency.

To support our findings, Hendarto et al. [55] found that there is no significant difference in the biomechanics data of Boccia underhand throws between male and female athletes. Comparing the body kinematics of the underhand throw movement, female players have a greater swing angle (backswing and frontswing) than male players. The authors highlighted that although it is not significant, females also had more power and a slower follow-through, while the tilt of the upper body when throwing showed that males tilted their body more when throwing the ball. In detail, they observed that the average distance of the ball from the target in female athletes is closer to the target than in male athletes; namely, the average distance of the ball from the target in female athletes was 0.04 m while in male athletes was 0.10 m. Although not significant, the study underlined that the kinetic energy of the moving ball and momentum ball was greater in female athletes, resulting in a faster rolling ball speed.

Additionally, Gromeier et al. [56] showed that no statistically significant differences were found between genders in throwing accuracy. Male and female athletes showed similar movement patterns in humeral and forearm movements, but differed in trunk, stepping, and backswing actions. These authors’ results suggest that there are gender-specific differences in qualitative throwing performance, but not necessarily in quantitative throwing performance. We should highlight that throwing is a basic and complex motor skill, requiring body coordination with the upper extremities, and it involves fast arm movements [15]. The biomechanics in the underhand throw motion include friction, kinetic energy, power, and angle of movement [57]. Previously, Braendvik and Roeleveld [58] concluded that spasticity increases coactivation (concurrent activation of agonist and antagonist muscles around a joint) in the muscle antagonistic to the spastic one [59,60], although coactivation plays a minor role in muscle weakness in CP and does not limit force modulation [61]. Recently, Maselli et al. [62] also indicated that throwing accuracy is strongly associated with very precise finger release times concerning arm kinematics.

From our perspective, most of the Boccia athletes participating in these international-level events have undergone a long training process and have national and international experience before competing in EC, WC, and PG. These training and competitive moments provide the possibility of improving skills that are important for Boccia (namely, those associated with precision), as well as developing skills and strategies from a tactical point of view. For example, accuracy/precision requires information about the target and the target location so that the range can bring the limb toward the target [63]. Moreover, differences in movement may occur due to competitive experience, different training intensities, morphological profiles of each gender, and mastery of throwing techniques, assumptions following the results of the review that hand size and finger length are the main anthropometric factors related to throwing results. Besides that, players can achieve high throwing speeds without losing accuracy if they are involved in practice sessions [64]. These authors stressed that players with larger elbow angles and higher elbow displacement angles at the time of releasing the ball during the throw produce faster throws.

Thus, players with CP, although they require more time to perform the ball throwing, can adapt the moment to achieve the accuracy/precision required in international-level events. CP can essentially affect any body function or structure dependent upon its severity and type but is most typically associated with limitations in gross motor functioning, muscle spasticity, and sometimes with cognitive impairment [65]. Noteworthy, our results also showed differences in BC4, despite the observed little variance. This FC is related to players with other severe physical disabilities; players in BC4 have severe locomotor dysfunction of all four extremities as well as poor trunk control. They are diagnosed with conditions of non-cerebral origin, contrary to BC1, BC2, and BC3 classified as players with CP [66]. In our perspective, these factors may influence the performance in Boccia, and consequently, the balance of the partials, because BC4 athletes often lack strength, have progressive disabilities, and use an underhand, pendulum swing to release the ball. It should also be noted that they are not eligible for assistance, contrary to BC3, who normally use a ramp and a male or female assistant.

In our perspective, the role of the assistant and the use of the ramp requires deeper research because based on our results, BC3 was the FC with a higher p-value and a lower association level, indicating fewer differences in the game of Boccia. Crozier et al. [67] stressed that it is fair to say that the literature on perceptual-motor skills is rather mixed, and that throwing is a complex coordinative skill that can have different goals: throwing may aim to achieve maximum speed or distance or may aim to hit a target with high accuracy, and strength requirements depend on the distance to the target. Previously, Barnett et al. [68] reported that while locomotor skills did not show gender differences, boys were significantly more proficient in object control skills than girls, as exemplified in overhand throwing. McKay et al. [69] reported that after age 10, males performed better in gross motor skills, while females performed better in fine motor function. These pieces of evidence, associated with the training level of Boccia players participating in international-level events, explain the tendency for the Boccia partials to be closely disputed. In BC4 we found a high probability of Boccia partials won by female players to be considered adjusted (1–2 ball difference), and in contrast, a higher probability of balance in partials won by male Boccia players (3–4 ball difference). In both cases, the ball difference was short.

Gender equality is a challenge in Boccia, a parasport where globally the number of female athletes continues to be considerably lower compared to male, despite the number of female athletes, considering all parasports, has noticeably increased (more than tripled between 1984 and 2016 [70]. This was recently supported in Tokyo 2020, which took place in 2021 due to the COVID-19 pandemic, in which only male players won medals considering the individual FC. We could not analyse the partials in Boccia in Tokyo 2020 because only game results are available. Still, in that particular event, from the 114 participants, 73 were men and only 41 were women, in line with the tendency observed by Ferreira et al. [70] of growing female participation in PG in 2012 (76 male and 27 female) and in 2016 (73 male and 33 female). In Paris 2024, for the first time in 40 years, men and women had their own individual competitions, designated as a “more gender-equal” format.

As people with physical disabilities themselves have demonstrated in the past and currently every day, we must not forget that they are people with great abilities who want to participate, on equal terms, in PA. As active members of the society to which they belong, they have the right to live as independently as possible and with the highest possible (QoL). A society that does not recognize the value of people with functional diversity will lose all the potential they have to offer [71]. Studies can be found in the literature that examine different types of barriers and their multiple classifications, such as social barriers, including discrimination and stigma; psychological barriers, including low levels of self-esteem and lack of motivation; and physical barriers, such as lack of access to adapted facilities and equipment [72,73]. Moreover, there are interpersonal barriers, such as lack of support or disapproval from others [29]. It is worth noting the existence of the differentiation in types of barriers according to gender [74]. In this sense, although from a performance perspective in Boccia this study suggests that gender separation is not supported by results in international-level events, strategies to promote more women’s participation in Boccia seem adequate aiming motivation to practice, more opportunities for recognition of performance (classifications, medals) and promotion of (QoL) in people with disabilities.

A clear limitation associated with Boccia is that the literature draws attention to the lack of sufficient research on the important components of training para-athletes or adapted sports [75]; besides, research in this adapted sport is scarce. We should, at this point, again indicate the inexistence of partial results in other international-level Boccia events between 2012 and 2018 also as a limitation. Moreover, the results of this study should not be extrapolated to other contexts because it is related to elite athletes, representing their countries in international-level Boccia events such as EC, WC, and PG, and the large majority of Boccia players are involved in adapted sports in institutions, clubs, or at a recreational level.

From our perspective, there is still little understanding of performance indicators in Boccia, as well as concerning the effects of training on performance. In this regard, it should be borne in mind that future research could focus on specific details such as the tactical aspect of the game (playing individually, in teams or pairs), the influence of the assistants in the BC3 classes, or using inertial devices to evaluate internal and external load variables of the players during the throw of the balls in the Boccia game. Future studies could consider the analysis of games instead of partials to be able to include a greater number of competitive events without information available regarding the partials in Boccia. Moreover, we also consider it pertinent to carry out a more detailed analysis to substantiate competition results, namely, potential confounding factors such as differences in training regimens, athlete experience, or age distributions.

## 5. Conclusions

This study revealed that female participation and performance in Boccia increased in international-level events between 2016 and 2018, following the uptrend in men, which showed a tendency to stabilize between 2017 and 2018. Interestingly, we did not find differences in the 2012–2018 period considering the players’ gender and the type of partials in BC1, BC2, and BC3. These results emphasize that, based on performance, both men and women can play together, which has been the reality since the introduction of Boccia in PG 1984 but was recently changed, since men and women were again separated in Rio de Janeiro 2022 WC and Paris 2024 PG.

From our point of view, it is understandable the recent perspective of separating genders in Boccia if the focus is toward more female participation and greater opportunity to recognize female athletes (standings/medals), focusing on gender equality, effective participation, and women’s empowerment. The differences found in BC4 (despite the low association level), and the specific characteristics of athletes integrated in this class, shed light regarding the need to deepen the research in this adapted sport, in which athletes are grouped based on their FC. Our results also underline the importance of more deeply studying BC3, which is associated with particularities that distinguish it from other classes, namely, the use of ramp and the potential influence of the assistant in the performance and results in Boccia. The regulatory measures determined by organizations in adapted sports can influence motivation to practice, and in this sense, opportunities to promote (QoL) in people with disabilities.

## Figures and Tables

**Table 1:** Classification of type of partial considering the number of ball differences.

	Type of Partial
Adjusted	(1–2 ball difference)
Balanced	(3–4 ball difference)
Unbalanced	(5–6 ball difference)

**Table 2:** Boccia partials considering the FC in four international-level events.

FC	London (PG 2012)	Rio de Janeiro (PG 2016)	Póvoa do Varzim (EC 2017)	Liverpool (WC 2018)	Total
BC1	84	127	137	252	600
BC2	124	124	211	252	711
BC3	104	127	263	250	744
BC4	72	125	209	247	653
Total	384	503	820	1001	2708

FC: Functional Classification; PG: Paralympic Games; EC: European Championships; WC: World Championships.

**Table 3:** Descriptive analysis of the winning partials considering the different Boccia FC in four international-level events.

	Partials (*n* = 2708)					
FC	Male		Female		Total	
	*n*	%	*n*	%	*n*	%
BC1	509	84.83	91	15.17	600	22.16
BC2	577	81.15	134	18.85	711	26.26
BC3	556	74.73	188	25.27	744	27.47
BC4	537	82.24	116	17.76	653	24.11

FC: Functional Classification.

**Table 4:** Influence of gender in Boccia type of partials (*n* = 2708 international-level partials).

Gender	Type of Partials						
	χ^2^	df	*p*	φc	*p*	Association Level	Observations
BC1	0.862	2	0.650	0.038	>0.05	-	No differences
BC2	4.641	2	0.099	0.081	>0.05	-	No differences
BC3	0.341	2	0.840	0.021	>0.05	-	No differences
BC4	8.446	2	0.015	0.114	<0.05	Low	Differences

χ^2^: Chi-square; df: degree of freedom; φc: Cramer’s Phi coefficient; Association level according to Crewson’s: Small (<0.100), Low (0.100–0.299), Moderate (0.300–0.499), and High (>0.500).

**Table 5:** Descriptive results and ASR considering the type of partial and gender in BC4.

Variables	Gender					
	Male			Female		
	*n*	%	ASR	*n*	%	ASR
BC4						
Adjusted	389	72.44	−2.9	99	85.34	2.9
Balanced	125	23.28	2.7	14	12.07	−2.7
Unbalanced	23	4.28	0.8	3	2.59	−0.8

*n*: frequency; %: percentage; ASR: ASR: Adjusted Standardized Residuals > |1.96|.

## Data Availability

The data supporting this study’s findings are available from the first and corresponding author, upon reasonable request.
